# Technical Aspects of Developing Chatbots for Medical Applications: Scoping Review

**DOI:** 10.2196/19127

**Published:** 2020-12-18

**Authors:** Zeineb Safi, Alaa Abd-Alrazaq, Mohamed Khalifa, Mowafa Househ

**Affiliations:** 1 Division of Information and Computing Technology, College of Science and Engineering Hamad Bin Khalifa University Qatar Foundation Doha Qatar; 2 Centre for Health Informatics, Australian Institute of Health Innovation Faculty of Medicine, Health, and Human Sciences Macquarie University Sydney Australia

**Keywords:** chatbots, conversational agents, medical applications, scoping review, technical aspects

## Abstract

**Background:**

Chatbots are applications that can conduct natural language conversations with users. In the medical field, chatbots have been developed and used to serve different purposes. They provide patients with timely information that can be critical in some scenarios, such as access to mental health resources. Since the development of the first chatbot, ELIZA, in the late 1960s, much effort has followed to produce chatbots for various health purposes developed in different ways.

**Objective:**

This study aimed to explore the technical aspects and development methodologies associated with chatbots used in the medical field to explain the best methods of development and support chatbot development researchers on their future work.

**Methods:**

We searched for relevant articles in 8 literature databases (IEEE, ACM, Springer, ScienceDirect, Embase, MEDLINE, PsycINFO, and Google Scholar). We also performed forward and backward reference checking of the selected articles. Study selection was performed by one reviewer, and 50% of the selected studies were randomly checked by a second reviewer. A narrative approach was used for result synthesis. Chatbots were classified based on the different technical aspects of their development. The main chatbot components were identified in addition to the different techniques for implementing each module.

**Results:**

The original search returned 2481 publications, of which we identified 45 studies that matched our inclusion and exclusion criteria. The most common language of communication between users and chatbots was English (n=23). We identified 4 main modules: text understanding module, dialog management module, database layer, and text generation module. The most common technique for developing text understanding and dialogue management is the pattern matching method (n=18 and n=25, respectively). The most common text generation is fixed output (n=36). Very few studies relied on generating original output. Most studies kept a medical knowledge base to be used by the chatbot for different purposes throughout the conversations. A few studies kept conversation scripts and collected user data and previous conversations.

**Conclusions:**

Many chatbots have been developed for medical use, at an increasing rate. There is a recent, apparent shift in adopting machine learning–based approaches for developing chatbot systems. Further research can be conducted to link clinical outcomes to different chatbot development techniques and technical characteristics.

## Introduction

Chatbots are systems that are capable of conversing with users in natural language in a way that simulates the interaction with a real human. The development of chatbots has captured the attention of researchers for a long time. Eliza [1] was one of the earliest attempts at developing a conversational system. Since then, chatbot development has evolved to be an integral part of many application domains. The most prominent example is the use of chatbots as personal assistants such as Apple’s Siri and Google Assistant. Chatbots have also been developed and are being used in different application areas, such as marketing and to provide different types of services [2,3].

Since the early years of their development, people adopted different techniques in designing and developing chatbots. In recent years, with the increasing adoption of machine learning and artificial intelligence methods in different application domains, state-of-the-art methods in dialogue generation and dialogue management are increasingly using machine learning and deep learning methods [4-6].

The medical field is an application area where chatbots are increasingly being adopted as a tool to facilitate access to information from the patient side and reduce the load on the clinician side. Many commercial chatbot applications that are available as web or mobile applications have been developed for interacting with patients. Some examples of health care chatbots are OneRemission1, which was developed to help cancer survivors; Babylon Health, which is a symptom checker; and Wysa [7], which is a mental health chatbot that interacts with user to help with signs of anxiety and depression.

It is important to know the current state of different methods and techniques that are being employed in developing chatbots in the medical domain for many reasons. Conducting such a survey will help researchers in the future identify the different methods that have been used and to build on the existing approaches to develop more intelligent chatbots that provide a more natural experience to the user. It is also important to see where the current state of chatbot development stands with respect to developing chatbots for other applications. Therefore, in this work, we conducted a scoping review of the available literature on chatbot development in the medical field and constructed and identified the main components involved in chatbot development, as well as a description of techniques used in developing each of the identified components. The main objective of this study was to explore technical aspects and development methodologies associated with chatbots used in the medical field to explain best methods of development and support chatbot development researchers in their future work.

## Methods

This study follows a scoping review methodology. Specifically, it follows the PRISMA extension of scoping reviews [8]. In this section, we explain the details of the adopted methodology to conduct the review. The PRISMA extension for scoping reviews is presented in Multimedia Appendix 1.

### Search Strategy

#### Search Sources

We searched 8 databases (IEEE, ACM, Springer, ScienceDirect, Embase, MEDLINE, PsycINFO, and Google Scholar) to collect studies relevant to the topic. For Google Scholar, we only used the first 100 results from each search string, as Google Scholar returns the most relevant results belonging to each search query first. The search was conducted between September 9, 2019 and September 13, 2019. For the forward reference list checking, we used the cited-by functionality of Google scholar. We also checked the reference list of the included studies to review the backward reference list.

#### Search Terms

We used 2 different sets of search terms to search the databases. The search term set was decided based on the type of studies indexed by the database. For databases that mainly indexed studies in the medical field (Embase, MEDLINE, PsycINFO), we relied on keywords that are strictly related to the intervention (eg, chatbot, chatterbot, conversational agent, conversational bot). For databases with no specific application domain, we resorted to using keywords that are related to the medical domain (eg, health, medical, illness, disease, disability) in addition to the intervention-related words. The search strategy used for searching the databases is presented in Multimedia Appendix 2.

### Study Eligibility Criteria

The purpose of this work was to review the technical aspects of developing text-based chatbots in the medical field. Therefore, for a study to be considered, it had to satisfy the following criteria: describe a chatbot application, the chatbot must be developed for a medical application (eg, management, diagnosis, counseling), the input or output modality of the chatbot must be text, and the technical details of how the input is processed and output is produced must be mentioned. Studies that used a Wizard of Oz experiment design were excluded. In addition, some restrictions on the language of the study and publication type were enforced. Only studies that were published in English were included, and only peer-reviewed articles, conference papers, thesis, dissertations, and industrial and academic reports were considered.

### Study Selection

The study selection was conducted in 2 stages. Title and abstract screening was followed by a full-text screening stage. Both stages were conducted by 2 reviewers. The first reviewer, ZS, performed the screening of the full set of articles. Due to time constraints, the second reviewer, AA, reviewed a randomly selected set of 50% of the articles. Disagreements between the reviewers were resolved by a third reviewer, MH. To evaluate the interrater agreement, we used Cohen kappa [9]. The reviewers had substantial agreement in both stages, with a kappa measure of 0.74 and 0.67 in the first and second stages, respectively.

### Data Extraction

The data extraction was conducted by ZS following a preset form. The data extracted pertained to the metadata of the included studies as well as the different technical modules of interest in the study, such as the text understanding module, text generation module, and method of linking these modules. The data extraction form is shown in Multimedia Appendix 3.

### Study Quality Assessment

As this is a scoping, not a systematic, review, no study quality assessment was conducted for the purposes of this work.

### Data Synthesis

We used a narrative approach to synthesize the different reported results. We included a description of the included studies and a description of the different techniques used for the development of the chatbots.

## Results

### Search Results

**Figure 1:**
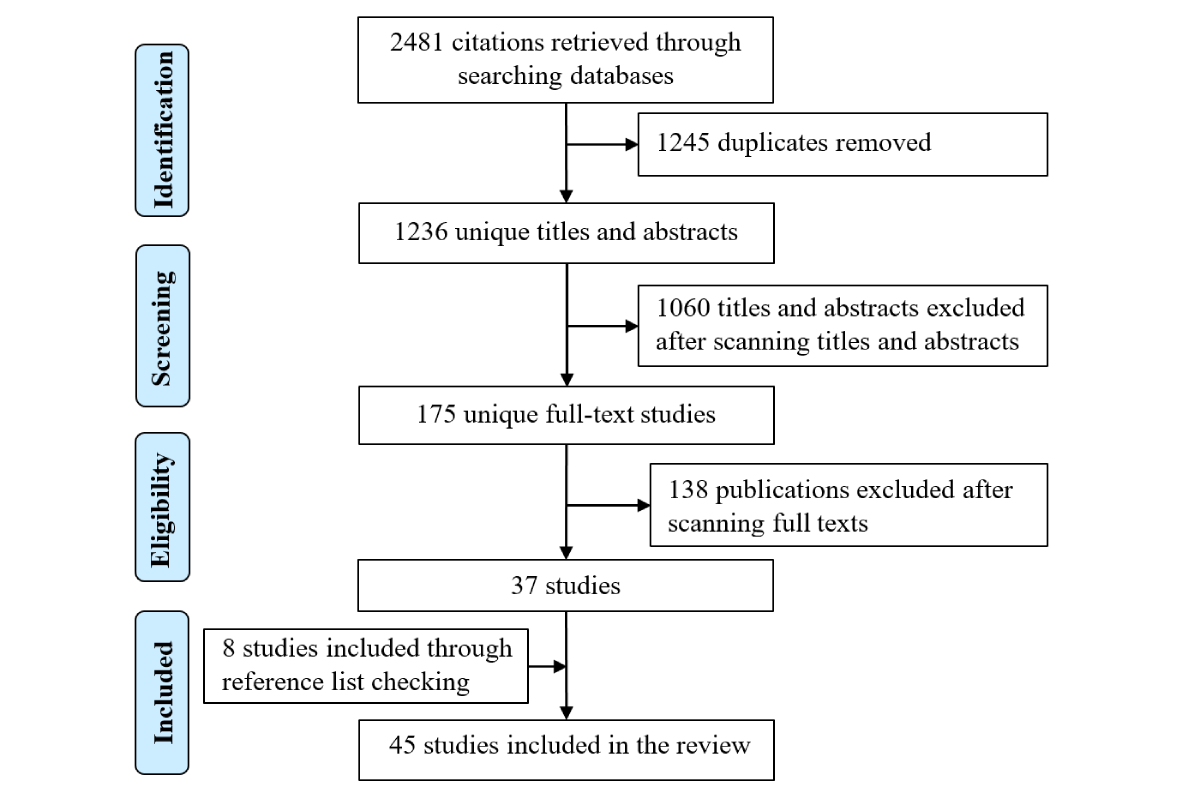
Study selection process.

Figure 1 summarizes the process that was followed to select the studies. Of the 2481 total studies returned after searching the databases, 1245 were duplicated. After removing the duplicates, 1236 studies remained and were screened based on title and abstract. After the title and abstract–based screening, 1060 studies were removed for the following reasons: not describing a chatbot (n=840), not containing technical details of the chatbot implementation (n=4), not belonging to a medical application (n=172), not containing text understanding or text generation (n=5), not written in the English language (n=8), and non-peer–reviewed publications (n=31). After the full-text screening phase, 138 additional studies were removed for the following reasons: not describing a chatbot (n=35), not containing technical details of the chatbot implementation (n=56), not belonging to a medical application (n=3), not containing text understanding or text generation (n=27), not written in the English language (n=1), and non-peer–reviewed publications (n=16). After performing forward and backward reference checking, 8 additional studies were included. The total number of included studies was 45.

### Description of Included Studies

The included studies were published between the years 2009 and 2019, with 80% (36/45) of the studies published in the last 5 years, in the years 2015-2019. Conference papers were the most common publication type (23/45, 51%), followed by journal papers (19/45, 42%) in addition to 2 magazine articles and 1 thesis. The most common country for publications was the United States, with 8 studies, followed by Australia, India, and Switzerland, with 4 studies each. Multimedia Appendix 4 shows the characteristics of each included study.

### Description of Chatbots

The total number of chatbots assessed in this study was 45. The chatbots were developed for different diseases and to fulfill different purposes. Table 1 shows the different categories and number of chatbots developed in each category. The most common category was “General Health,” which includes chatbots designed for health educational or counseling purposes for general health conditions. These chatbots can also provide information on general medical services such as disease diagnosis based on patient-given symptoms. Some provide patients with discharge information before leaving the hospital. The second most common type of chatbot is those developed for mental health purposes, followed by those developed for specific diseases, such as diabetes, cancer, autism, heart disease, and asthma.

An important factor for the technical aspects of developing chatbots is the language that the chatbot uses for communication. The majority of chatbots communicate in English (23/45, 51.1%), 4 in German, 3 in Chinese, 2 in Arabic, 2 in Korean, 1 in Thai, 1 in Spanish, and 1 in Russian; 8 studies did not mention the language. The chatbots operated as either standalone applications (n=17) or web applications (n=20), while the remaining studies did not mention the application type (n=8).

**Table 1:** Target diseases for chatbot development in the included studies (n=45).

Disease/condition	Count	Percentage (%)	Studies
General health	21	47	[10-31]
Mental health	15	33	[32-45]
Diabetes	2	4	[46,47]
Cancer	2	4	[48,49]
Autism	2	4	[50,51]
Heart disease	1	2	[52]
Asthma	1	2	[53]
HIV	1	2	[54]

### Chatbot Implementation

#### Overview

**Figure 2:**
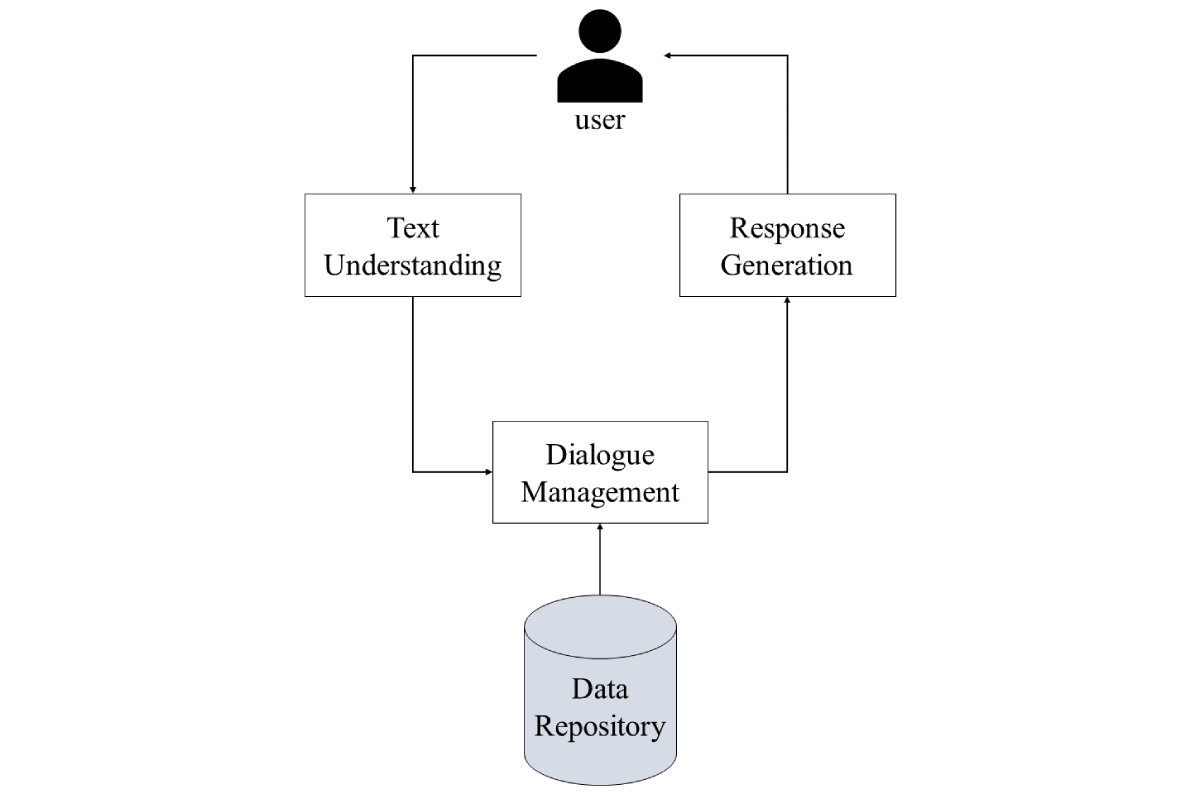
High-level chatbot architecture.

The chatbots in the included studies consisted of 4 main modules: text understanding module, dialog manager, text generation module, and database layer that holds the various types of information needed for chatbot training and function. Figure 2 shows a high-level architecture of chatbot development and the relationship between the different modules that constitute it. The following subsections highlight the findings in terms of how each of the previously mentioned components is implemented.

#### Text Understanding

The text understanding module is the module with which the user directly interacts. The function of the text understanding module is to extract meaning from the user input before a specific answer is generated. In the included studies, 7 methods were used for text understanding. Pattern matching methods were the most common, used by 18 of the 45 chatbots (40%). Many text understanding approaches can fall under pattern matching. The different pattern matching methods reported in the included studies are as follows: keyword matching or string matching, where specific keywords or strings of the user input are matched to scripts stored in the database, which was used in 11 studies [10-15,32,33,46,48,50], or Extensible Markup Language (XML) variants, such as Artificial Intelligence Markup Language (AIML), which were used in 7 studies [16,17,34-37,47]. The second most popular approach was the use of machine learning methods for text understanding, which was used in 6 studies. Most of these studies used supervised machine learning algorithms, including decision trees and random forests. These include groups of decision trees, where each tree gives a classification to the text, in the form of voting, while the forest chooses the classification having the most votes over all the trees in the forest. Machine learning training materials were sometimes based on real health data extracted and anonymized from hospital information systems and electronic health records. In other instances, chatbots were trained on billions of words extracted from Google News datasets [18,19,38,39,49,51]. Another 6 studies relied on web services such as Dialogflow by Google. Web services are usually provided through a computer server that responds to requests through ports over a network, such as the internet, to solve specific domain problems over the web. They can also use a mixture of machine learning and rule-based methods to produce a customizable chatbot implementation. Three studies used natural language processing–based approaches, such as named entity recognition, to extract meaning from user input. To develop the chatbot, 2 of the studies used a rule-based approach, where the chatbot is operated by either a set of IF-THEN rules or a state-based system. A few of the studies relied on a fixed input method, where the user selects the input from a list of possible inputs. Fixed input chatbots do not technically need to go through a text understanding module as the system does not need to interpret the user input. The input needs only to be directed to the dialogue manager. Some studies proposed a few hybrid text understanding approaches. Hybrid approaches use a combination of 2 or more of the previously mentioned methods for text understanding. Table 2 summarizes the different text understanding methods proposed and the studies that fall under each method.

**Table 2:** Text understanding methods used in the studies (n=45).

Text understanding method	Studies
Pattern matching	[10-17,32-37,46-48,50]
Machine learning	[18,19,38,39,49,51]
Web services	[20-22,40,53,54]
Fixed input	[23-25,41,42]
Natural language processing	[26-28]
Hybrid	[43,44,52]
Rule-based	[29,30]
Not mentioned	[31,45]

#### Dialogue Management

In the reported studies, the input to the dialog management module is the processed user free-text input provided by the text understanding module. The dialog management module controls the different aspects of the conversation and links each user input to an appropriate output. In the included studies, 2 main types of dialog management techniques were reported: static dialog management and dynamic dialog management. In the case of static dialog management, user input is matched directly to the appropriate output using a pattern matching algorithm (25/45) or using a set of rules (7/45). In the case of dynamic dialogue management, the context of the conversation changes based on specific user input characteristics. The context switching can be done by training machine learning algorithms to identify the context from the user input (5/45) or using a web service for intent identification (4/45). Table 3 summarizes the different dialogue management methods and the studies that correspond to each method.

**Table 3:** Dialogue management methods in the included studies (n=45).

Dialogue management method/platform	Studies
Pattern matching algorithms	[10,12,14-19,22,25,28,32-38,42,45-50]
Rule-based methods	[11,26,27,29-31,52]
Machine learning	[39,40,43,44,51]
Web-based	[20,21,53,54]
Not mentioned	[13,23,24,41]

#### Data Management

Most of the chatbots in the included studies contained one or more of the 3 data repository types identified. Most of the included studies kept a medical knowledge repository (28/45, 62%). The medical knowledge repository contains medical information related to the application domain of the developed chatbot. The medical knowledge source can be local, such as obtaining it from medical personnel, or it can be collected from online sources such as Wikipedia or other websites. The medical knowledge can be presented to the user in the context of educational chatbots (17/45) [12-14,19,23-25,27,32,34, 41,46,48-51,53], or it can be used to train machine learning algorithms (5/45) [39,40,43,44,51]. Many of the developed approaches store users’ data and use the data to customize the chatbot response and improve its functionality (11/45). Conversation scripts are the third-data repository type, and they are usually kept by chatbots that use pattern matching as a text understanding or text generation modality (5/45). A few studies did not mention the type of data stored (9/45). Table 4 summarizes the different database types and studies that reported keeping each database type.

**Table 4:** Database types in the included studies (n=45).

Database type	Studies
Medical knowledge database	[10,11,13,15-17,20,22-24,26-28,31-33,36-42,47,48,50-53]
User information database	[11,13,18,21,25,39,40,45,46,50,53]
Conversation scripts	[11,12,14,15,19]
Not mentioned	[21,29,30,34,35,43,44,49,54]

#### Text Generation

The text generation module provides output to the user. Text generation in the included studies was done using one of 2 methods: fixed output or generated output. The fixed output methods search the database for the most appropriate output to a user input and present it to the user. The generated output method relies on machine learning to generate original natural language output that is produced by the machine learning algorithm. Chatbots in most of the included studies provide fixed output that is extracted from the database with the exception of those in [25,39,51], which provide output that is generated using machine learning and deep learning methods, and [43], which proposes a hybrid approach that can provide fixed and generated outputs. A few studies did not report the output generation method, or it was not applicable as the output modality was not text [23,24,41,42,45].

## Discussion

### Principal Findings

A general architecture was identified and reported to summarize the technical aspects of chatbot development. The main components of chatbots, as well as the way these components are linked, are reported. Chatbots typically consist of 4 main components: text understanding module, dialogue management module, data management layer, and text generation module.

The most common design method employed in developing chatbots is pattern matching for text understanding and response generation. Machine learning and generative methods are among the least commonly used methods for the development of chatbots in the medical domain. This can be attributed to 2 main reasons. The first reason for relying on pattern matching approaches more than those based on machine learning is that pattern matching methods are more reliable in practice because they produce exact responses to well-defined queries, resulting in fewer mistakes. Machine learning–based methods usually produce different types of errors, which cannot be tolerated in medical applications. The second reason for this trend is the rapid development in the state of the machine learning field over the past few years and the increase in the robustness of its methods, especially with the emergence of deep learning. While older methods relied on rule-based chatbots and pattern matching algorithms, all the proposed methods that rely on machine learning for text understanding and response generation were proposed between the years 2017 and 2019. Another reason for the possible lack of using machine learning methods could be the fact that machine learning–based approaches need to be trained using large amounts of domain-specific data, which might be scarce and difficult to access in the medical field. Overall, machine learning approaches and algorithms were better suited for developing chatbots used for specific medical conditions, such as mental health and autism, while the rule-based approaches were better suited for developing chatbots used for general medical purposes. On the other hand, pattern matching methods and algorithms were more broadly used in developing chatbots used for both special and general medical conditions.

In terms of data management, the developed chatbots kept track of 3 different types of databases: medical knowledgebase including a library of medical facts, user information database including details about users’ demographics and their preferences, and dialogue script database including all possible entries of conversational text responding to users. The type of database kept depends on the chatbot type and target functionality. Educational chatbots usually keep a medical knowledgebase. Chatbots that use context switching based on user emotions usually keep a user information database.

Most of the developed chatbots used English as the language of communication with the users, while other languages such as German, Chinese, and Arabic were less common. This is consistent with the fact that most of the publications originated in the United States, followed by Australia, where the first language is English.

### Strengths and Limitations

#### Strengths

This review focused generally on chatbots in the medical field, without specifying the field of application, which makes it more comprehensive than previously conducted reviews [55]. Other reviews that included different medical applications [56] presented a general taxonomy of conversational agents, while we presented a more granular description of the development techniques of each component. Searching more libraries from different application domains allowed us to include more chatbots in our study than that of Montenegro et al [56]. This is why we excluded Wizard of Oz studies, while they were used in the previous reviews. Two similar systematic review studies were conducted earlier in 2018 and 2019 [57,58]. The first study reviewed the applications and evaluation measures of chatbots and conversational agents, while the second paper provided a critical review of the tasks involved in natural language understanding and machine learning of chatbot systems used in the medical domain. Neither of the 2 papers discussed the technical aspects and development methodologies of the chatbots used in the medical domain.

#### Limitations

This review only focused on text-based chatbot applications, where either the input or output modality is written. This excludes studies where the input or output modalities are spoken or visual, as well as robotics and telephone-based methods. This choice was made because we wanted to focus on text processing techniques rather than image or voice processing, as speech-to-text technologies can also introduce errors and another layer of complexity to chatbot development.

We enforced some constraints on the type of publications that were included in the current review. These constraints might have led to missing a portion of developed chatbots that have been published in other research venues, such as workshops, book chapters, and conference abstracts. Furthermore, limiting the search to papers published in English could also have led to missing some chatbots that were developed for communication in other languages and published in their own language. For example, we did not include papers published in Chinese or Arabic that discuss chatbots communicating in these languages.

This review focused on the development process of chatbots without considering the impact of these methods on patients. For this reason, some of the implementations in some of the included studies might be feasible from a technical point of view, but this does not necessarily mean they are effective from a medical point of view.

### Practical and Research Implications

#### Practical Implications

This paper reports the technical aspects of developing chatbots in the medical field. This review can be used to identify the most common development approaches by specialists to help them narrow down their options and make a decision on which development approach is the most appropriate for their applications.

The reported results show that most of the developed chatbots communicate with users in the English language. While a few attempts to design and develop chatbots in other languages exist, more work needs to be done in this regard, especially for languages that are spoken by a large portion of the world’s population, such as Chinese and Arabic.

Even though dynamic dialogue management provides a more natural user experience, most developed systems rely on static dialogue management methods. Changing the dialogue context based on user emotions or by detecting topic changes in user input are important aspects to be considered in chatbot development.

The use of machine learning and artificial intelligence methods in the development of conversational agents in different application areas has recently increased. The rate of adoption of machine learning–based methods in developing chatbots is still relatively low, even though it has been increasing in recent years. Supervised machine learning algorithms seem to better suit the development of chatbots for special medical conditions and diseases, while rule-based methods are being used more for developing chatbots used for general medical purposes. Machine learning methods allow the development of more intelligent agents that can provide a more realistic user experience, by providing a better text understanding experience, including more dynamic and flexible dialogue management, and generating a wider range of responses.

#### Research Implications

As the purpose of this review was to survey the technical aspects of chatbot development, the clinical results of performing clinical trials were not considered. Further reviews linking the different development techniques used to the clinical outcomes of the chatbot developed are possible and recommended.

More openness and a wider adoption of state-of-the-art methods in dialogue management [4], text understanding [5], and text generation [6] methods in the literature can really benefit the development of conversational agents in the medical field.

It is worth noting that the technical aspects of developing chatbots were not always clearly mentioned in the studies. The devised architecture is a general one that does not necessarily apply to every developed chatbot. One or more component might be omitted, and the chatbot might still function properly.

### Conclusion

In the scope of this review, we analyzed the technical aspects of developing 45 text-based chatbots for the purpose of performing different medical interventions. The most common language used for chatbot communication is English. Chatbots typically contain 4 main components: text understanding module, dialogue manager, database layer, and text generation module. The most common technique for developing chatbots is using a string matching algorithm and a set of scripts that contain sample inputs and outputs. Judging from the publication years of the different studies, we can conclude that chatbots are becoming increasingly popular for medical application, especially when it comes to mental health. The adoption of machine learning and artificial intelligence–based techniques has recently increased. Some development approaches are better suited than others for developing chatbots for specific medical conditions rather than general medical conditions. Future studies can be conducted to link the development techniques of chatbots to their clinical outcomes. It is important to conduct more in-depth systematic reviews on the effectiveness of chatbots in supporting and enhancing positive clinical outcomes. We need to understand and correlate different technical criteria and development methodologies to different levels of chatbots acceptance, utilization, and clinical effectiveness. Discussing the pros and cons of each chatbot system has also been left to future supplementary studies, to compare advantages and disadvantages of each chatbot system and link these to their postimplementation clinical outcomes.

## Appendix

Multimedia Appendix 1 Preferred Reporting Items for Systematic reviews and Meta-Analyses extension for Scoping Reviews (PRISMA-ScR) checklist.

Multimedia Appendix 2 Search strategy.

Multimedia Appendix 3 Data extraction form.

Multimedia Appendix 4 Chatbot description.
