# Supplementary material for: Technical Aspects of Developing Chatbots for Medical Applications: Scoping Review
Source: J Med Internet Res. 2020 Dec 18;22(12):e19127. doi: 10.2196/19127 (PMC7775817; doi:10.2196/19127)
Supplement: Multimedia Appendix 1 [file jmir_v22i12e19127_app1.pdf]

Preferred Reporting Items for Systematic reviews and Meta-Analyses extension for Scoping Reviews (PRISMA-ScR) Checklist

SECTION	ITEM	PRISMA-ScR CHECKLIST ITEM	REPORTED ON PAGE #
**TITLE**			
Title	1	Technical Aspects of Developing Chatbots for Medical Applications: A Scoping Review.	1
**ABSTRACT**			
Structured summary	2	**Background:** Chatbots are applications that can conduct natural language conversations with users. In the medical field, chatbots have been developed and used to serve different purposes. They provide patients with timely information that can be critical in some scenarios, such as access to mental health resources. Since the development of the first chatbot, ELIZA in the late 1960s, much efforts followed to produce chatbots for various health purposes developed in different ways.<br><br>**Objective:** This study aims to review previous studies exploring different technical aspects used for developing text-based chatbots for healthcare.<br>Methods: We searched for relevant articles in 8 databases (IEEE, ACM, Springer, ScienceDirect, Embase, MEDLINE, PsycINFO and Google Scholar). We also performed forward and backward reference checking of the selected articles. Study selection was performed by one reviewer and 50% of the selected studies were randomly checked by a second reviewer. A narrative approach was used for result synthesis. Chatbots were classified based on the different technical aspects of their development. The main chatbot components were identified in addition to the different techniques for implementing each module.<br><br>**Results:** The number of retrieved publications in our original search was 2481, out of which we identified 45 studies that matched our inclusion and exclusion criteria. The most common language of communication between users and chatbots was English (n=23). Four main modules were identified, the text understanding module, the dialog management module, the database layer and the text generation module. The most common technique for developing the text understanding and dialogue management are pattern matching methods (n=18 and n=25 respectively). The most common text generation is fixed output (n=36). Very few studies relied on generating original output. Most studies kept a medical knowledge base to be used by the chatbot for different purposes throughout the conversations. A few studies kept conversation scripts and collected user data and previous conversations.<br><br>**Conclusions:** Many chatbots have been developed for medical use, with an increasing rate. There is an apparent shift in adopting machine learning based approaches for developing chatbot systems in recent years. Further research can be conducted to link clinical outcomes to	2

SECTION	ITEM	PRISMA-ScR CHECKLIST ITEM	REPORTED ON PAGE #
		different chatbot development techniques and technical characteristics..	
**INTRODUCTION**			
Rationale	3	It is important to know the current state of different methods and techniques that are being employed in developing chatbots in the medical domain for many reasons. Conducting such a survey will help researchers in the future identify the different methods that have been used and to build on the existing approaches to develop more intelligent chatbots, that provide a more natural experience to the user. It is also important to see where the current state of chatbot development stands with respect to developing chatbots for other applications.	3
Objectives	4	In this work we conducted a scoping review of the available literature on chatbot development in the medical field and constructed and identified the main components involved in chatbot development, as well as a description of techniques used in developing each of the identified components. The main objective of this study is to explore technical aspects and development methodologies associated with chatbots successful implementation and use in the medical field.	3
**METHODS**			
Protocol and registration	5	This study follows a scoping review methodology. Specifically, it follows the PRISMA extension of scoping reviews. https://www.acpjournals.org/doi/10.7326/M18-0850	5
Eligibility criteria	6	The purpose of this work is reviewing the technical aspects of developing text-based chatbots in the medical field. Therefore, for a study to be considered, it must satisfy the following criteria: describe a chatbot application, the chatbot must be developed for a medical application (management, diagnosis, counseling etc.), the input and/or the output modality of the chatbot must be text, the technical details of how the input is processed and the output is produced must be mentioned. Studies that use a Wizard of Oz experiment design were excluded. In addition, some restrictions on the language of the study and publication type were enforced. Only studies that were published in English were included, and only peer-reviewed articles, conference papers, thesis, dissertations, and industrial and academic reports were considered.	5
Information sources*	7	Eight databases were searched to collect studies relevant to the topic (IEEE, ACM, Springer, ScienceDirect, Embase, MEDLINE, PsycINFO and Google Scholar). For Google Scholar we only used the first 100 results for each search string, as Google Scholar returns the most relevant results belonging to each search query first. The search was conducted between September 9th and September 13th of 2019. For the forward reference list checking, we used the cited by functionality of Google scholar. We also checked the reference list of the included studies to review the backward reference list.	5
Search	8	Search strategy for PubMed (MEDLINE):	5

SECTION	ITEM	PRISMA-ScR CHECKLIST ITEM	REPORTED ON PAGE #
		((Chatbots[Title/Abstract]) OR (talkbot[Title/Abstract]) OR (IM Bot[Title/Abstract]) OR (Interactive Agent[Title/Abstract]) OR (Conversation Entity[Title/Abstract]) OR (Conversation Agent[Title/Abstract])) AND ((Health[Title/Abstract]) OR (Healthcare[Title/Abstract]) OR (Medical[Title/Abstract]) OR (Hospital[Title/Abstract]) OR (Clinical[Title/Abstract]) OR (Clinic[Title/Abstract]) OR (Disease[Title/Abstract]) OR (Illness[Title/Abstract]) OR (Disability[Title/Abstract]))	
Selection of sources of evidence†	9	Eight databases were searched to collect studies relevant to the topic (IEEE, ACM, Springer, ScienceDirect, Embase, MEDLINE, PsycINFO and Google Scholar). For Google Scholar we only used the first 100 results for each search string, as Google Scholar returns the most relevant results belonging to each search query first. The search was conducted between September 9th and September 13th of 2019. For the forward reference list checking, we used the cited by functionality of Google scholar. We also checked the reference list of the included studies to review the backward reference list.	5
Data charting process‡	10	The study selection was conducted in two stages. A title and abstract screening followed by a full text screening stage. Both stages were conducted by two reviewers. The first reviewer, ZS, performed the screening of the full set of articles. Due to time constrains, the second reviewer, AA, reviewed a randomly selected set of 50% of the articles. Disagreements between the reviewers were resolved by a third reviewer, MH. To evaluate the interrater agreement, we used Cohen's kappa.	6
Data items	11	The data extraction was conducted by ZS following a preset form. The data extracted pertained to the metadata of the included studies, as well as the different technical modules of interest to the study, such as the text understanding module, the text generation module, and the method of linking these modules.	6
Critical appraisal of individual sources of evidence§	12	As this is a scoping review not a systematic one, no study quality assessment was conducted for the purposes of this work.	6
Synthesis of results	13	We used a narrative approach to synthesize the different reported results. We included a description of the included studies, and a description of the different techniques used for the development of the chatbots.	6
**RESULTS**			
Selection of sources of evidence	14	Figure 1 summarizes the process that was followed for selecting the studies. The total number of studies returned after searching the databases was 2481 out of which 1245 were duplicated. After removing the duplicates, 1236 studies remained and were screened based on title and abstract. After the title and abstract based screening, 1060 studies were removed for the following reasons: not describing a chatbot (n=840), not containing technical details of the chatbot implementation (n=4), not belonging to a medical application (n=172), not containing a text understanding or a	7

SECTION	ITEM	PRISMA-ScR CHECKLIST ITEM	REPORTED ON PAGE #
		text generation (n=5), not written in English language (n=8), and non-peer reviewed publications (n=31). After the full text screening phase, 138 additional studies were removed for the following reasons; not describing a chatbot (n=35), not containing technical details of the chatbot implementation (n=56), not belonging to a medical application(n=3), not containing a text understanding or a text generation(n=27), not written in English language (n=1), and non-peer reviewed publications (n=16). Eight studies were included after performing forward and backward reference checking. The total number of included studies is (n=45).	
Characteristics of sources of evidence	15	Results section reports on groups of studies and classify them through 4 tables: Table 1: Target Diseases for chatbot development, Table 2: Text Understanding Methods, Table 3: Dialogue Management Methods, and Table 4: Database types.	8-11
Critical appraisal within sources of evidence	16	Critical appraisal of individual studies was not applicable to this review. We have clarified this in the manuscript.	8-11
Results of individual sources of evidence	17	Results section reports on groups of studies and classify them through 4 tables: Table 1: Target Diseases for chatbot development, Table 2: Text Understanding Methods, Table 3: Dialogue Management Methods, and Table 4: Database types.	7-11
Synthesis of results	18	Results section reports on groups of studies and classify them through 4 tables: Table 1: Target Diseases for chatbot development, Table 2: Text Understanding Methods, Table 3: Dialogue Management Methods, and Table 4: Database types.	7-11
**DISCUSSION**			
Summary of evidence	19	A general architecture was identified and reported to summarize the technical aspects of chatbot development. The main components of chatbots, as well as the way these components are linked are reported in this study. Chatbots typically consist of 4 main components, a text understanding module, a dialogue management module, a data management layer and a text generation module.<br>The most common design method employed in developing chatbots is using pattern matching for text understanding and response generation. Machine learning and generative methods are among the least commonly used methods for the development of chatbots in the medical domain. This can be attributed to two main reasons. The first reason for relying on pattern matching approaches a lot more than those based on machine learning, where pattern matching methods are more reliable in practice because they produce exact responses to well defined queries, resulting in less mistakes. While machine learning based methods usually produce different types of errors, which cannot be tolerated in medical applications [55]. The second reason for this trend is the rapid development in the state of the machine learning field over the past few years, and the increase in the robustness of its methods, especially with the emergence of deep	13

SECTION	ITEM	PRISMA-ScR CHECKLIST ITEM	REPORTED ON PAGE #
		learning. While older methods relied on rule-based chatbots and pattern matching algorithms, all the proposed methods that rely on machine learning for text understanding and response generation were proposed between the years 2017 and 2019. Another reason for the possible lack of using machine learning methods could be the fact that machine learning based approaches need to be trained using large amounts of domain specific data, which might be scarce and difficult to access in the medical field.<br>In terms of data management, the developed chatbots kept track of three different types of databases: a medical knowledgebase, a use information database and a dialogue script database. The type of database kept depends on the chatbot type and target functionality. Educational chatbots usually keep a medical knowledge base. Chatbots that use context switching based on user emotions usually keep a user information database.<br>Most of the developed chatbots used English as the language of communication with the users, while other languages such as German, Chinese and Arabic were less common. This is consistent with the fact that most of the publications originated in the United States of America followed by Australia, where the first language is English.	
Limitations	20	This review only focuses on text based chatbot applications, where either the input or output modality is written. This excludes studies where the input and/or output modalities are spoken or visual, as well as robotics and telephone-based methods. This choice was made because we want to focus on text processing techniques rather than image or voice processing as speech-to-text technologies can also introduce errors and another layer of complexity to the chatbot development.<br>We enforced some constraints on the type of publications that can be included in the current review. These constraints might have led to missing a portion of developed chatbots that have been published in other research venues, such as workshops, book chapter, and conference abstracts. Furthermore, limiting the search to papers published in English could also have led to missing some chatbots that were developed for communication in other languages and published in their own languages. For example, we have not included papers published in Chinese or Arabic language, which discuss chatbots communicating in these languages.<br>This review focuses on the development process of chatbots without considering the impact of these methods on patients. For this reason, some of the implementation of some of the included studies might be feasible from a technical point of view, but this does not necessarily mean they are effective from a medical point of view.	15
Conclusions	21	In the scope of this review, we analyzed the technical aspects of developing 45 text-based chatbots developed for the purpose of performing different medical interventions. The most common language used for chatbot communication is English. Chatbots typically contain 4 main	16

SECTION	ITEM	PRISMA-ScR CHECKLIST ITEM	REPORTED ON PAGE #
		components, a text understanding module, a dialogue manager a database layer and text generation module. The most common technique for developing chatbots is using string matching algorithm and a set of scripts that contain sample inputs and outputs. Judging from the publication years of the different studies, we can conclude that chatbots are becoming increasingly popular in the medical application, especially when it comes to mental health. The adoption of machine learning and Artificial intelligence-based techniques is increasing in the past few years. Future studies can be conducted to link the development techniques of chatbots to their clinical outcomes. Discussing the pros and cons of each chatbot system has also been left to future supplementary studies, to compare advantages and disadvantages of each chatbot system and link these to their post-implementation clinical outcomes.	
**FUNDING**			
Funding	22	This contribution was made possible by NPRP grant #12S - 0303- 190204 from the Qatar National Research Fund (a member of Qatar Foundation). The statements made herein are solely the responsibility of the authors.	16

JBI = Joanna Briggs Institute; PRISMA-ScR = Preferred Reporting Items for Systematic reviews and Meta-Analyses extension for Scoping Reviews.

* Where *sources of evidence* (see second footnote) are compiled from, such as bibliographic databases, social media platforms, and Web sites.

† A more inclusive/heterogeneous term used to account for the different types of evidence or data sources (e.g., quantitative and/or qualitative research, expert opinion, and policy documents) that may be eligible in a scoping review as opposed to only studies. This is not to be confused with *information sources* (see first footnote).

‡ The frameworks by Arksey and O'Malley (6) and Levac and colleagues (7) and the JBI guidance (4, 5) refer to the process of data extraction in a scoping review as data charting.

§ The process of systematically examining research evidence to assess its validity, results, and relevance before using it to inform a decision. This term is used for items 12 and 19 instead of "risk of bias" (which is more applicable to systematic reviews of interventions) to include and acknowledge the various sources of evidence that may be used in a scoping review (e.g., quantitative and/or qualitative research, expert opinion, and policy document).

From: Tricco AC, Lillie E, Zarin W, O'Brien KK, Colquhoun H, Levac D, et al. PRISMA Extension for Scoping Reviews (PRISMAScR): Checklist and Explanation. Ann Intern Med. 2018;169:467–473. doi: 10.7326/M18-0850.
