# Supplementary material for: Technical Aspects of Developing Chatbots for Medical Applications: Scoping Review
Source: J Med Internet Res. 2020 Dec 18;22(12):e19127. doi: 10.2196/19127 (PMC7775817; doi:10.2196/19127)
Supplement: Multimedia Appendix 2 [file jmir_v22i12e19127_app2.docx]

# Appendix 2: Search Strategy

Database(s): **Ovid MEDLINE(R)**1996 to September Week 2 2019 
Search Strategy:

| **#** | **Searches** | **Results** |
| --- | --- | --- |
| 1 | conversational agent*.tw. | 53 |
| 2 | conversational bot*.tw. | 1 |
| 3 | conversational system*.tw. | 4 |
| 4 | conversational interface*.tw. | 2 |
| 5 | chatbot*.tw. | 20 |
| 6 | chat bot*.tw. | 11 |
| 7 | chat-bot*.tw. | 0 |
| 8 | chatter bot*.tw. | 0 |
| 9 | chatterbot*.tw. | 19 |
| 10 | smart bot*.tw. | 1 |
| 11 | smartbot*.tw. | 3 |
| 12 | smart-bot*.tw. | 3 |
| 13 | virtual agent*.tw. | 80 |
| 14 | embodied agent*.tw. | 46 |
| 15 | virtual coach*.tw. | 25 |
| 16 | virtual human.tw. | 60 |
| 17 | 1 or 2 or 3 or 4 or 5 or 6 or 7 or 8 or 9 or 10 or 11 or 12 or 13 or 14 or 15 or 16 | 210 |

Database(s): **PsycINFO**2002 to September Week 2 2019 
Search Strategy:

| **#** | **Searches** | **Results** |
| --- | --- | --- |
| 1 | conversational agent*.tw. | 185 |
| 2 | conversational bot*.tw. | 2 |
| 3 | conversational system*.tw. | 13 |
| 4 | conversational interface*.tw. | 7 |
| 5 | chatbot*.tw. | 51 |
| 6 | chat bot*.tw. | 13 |
| 7 | chat-bot*.tw. | 13 |
| 8 | chatter bot*.tw. | 1 |
| 9 | chatterbot*.tw. | 7 |
| 10 | smart bot*.tw. | 1 |
| 11 | smartbot*.tw. | 1 |
| 12 | smart-bot*.tw. | 1 |
| 13 | virtual agent*.tw. | 192 |
| 14 | embodied agent*.tw. | 188 |
| 15 | virtual coach*.tw. | 48 |
| 16 | virtual human.tw. | 155 |
| 17 | 1 or 2 or 3 or 4 or 5 or 6 or 7 or 8 or 9 or 10 or 11 or 12 or 13 or 14 or 15 or 16 | 475 |

Database(s): **Embase**1996 to 2019 Week 40 
Search Strategy:

| **#** | **Searches** | **Results** |
| --- | --- | --- |
| 1 | conversational agent*.tw. | 70 |
| 2 | conversational bot*.tw. | 1 |
| 3 | conversational system*.tw. | 8 |
| 4 | conversational interface*.tw. | 4 |
| 5 | chat bot*.tw. | 17 |
| 6 | chatbot*.tw. | 45 |
| 7 | chat-bot*.tw. | 17 |
| 8 | chatterbot*.tw. | 0 |
| 9 | chatter bot*.tw. | 0 |
| 10 | smart bot*.tw. | 7 |
| 11 | smartbot*.tw. | 2 |
| 12 | smart-bot*.tw. | 7 |
| 13 | virtual agent*.tw. | 85 |
| 14 | embodied agent*.tw. | 39 |
| 15 | virtual coach*.tw. | 55 |
| 16 | virtual human.tw. | 302 |
| 17 | 1 or 2 or 3 or 4 or 5 or 6 or 7 or 8 or 9 or 10 or 11 or 12 or 13 or 14 or 15 or 16 | 470 |

| **Databases** | **Search strings** | **Hit** |
| --- | --- | --- |
| **ACM digital Library** | (("conversational agent*" OR "conversational bot" OR "conversational bots" OR "conversational system*" "chatbot*" OR "chat bot" OR "chat bots" OR "chat-bot" OR "chat-bots" OR "smartbot" OR "smartbots" OR "smart bot" OR "smart bots" OR "smart-bot" OR "smart-bots") AND (+Health Disorder* OR Disease* OR well-being OR illness OR medical OR medicine OR patient OR patients) | 87 |
| **IEEE Xplore** | (("Abstract":"conversational agent*" OR "conversational bot" OR "conversational bots" OR "conversational system*" OR "chatbot*" OR "chat bot" OR "chat bots" OR "chat-bot" OR "chat-bots" OR "smartbot" OR "smartbots" OR "smart bot" OR "smart bot" OR "smart-bot" OR "smart-bots" OR "relational agent" OR "relational agents" OR "agent based system" OR "agent based systems") AND ("Full Text Only":Health OR Disorder* OR Disease* OR well-being OR illness OR medical OR medicine OR patient OR patients)) | 319 |
| **Google Scholar** | ((“conversational agent*” OR “conversational bot*” OR "conversational system*" OR “conversational interface*” OR “chatbot*” OR “chat bot*” OR “chat-bot*”) AND (Health OR Disorder* OR Disease* OR patient* OR illness OR medical) | 100 |
