# Supplementary material for: Technical Aspects of Developing Chatbots for Medical Applications: Scoping Review
Source: J Med Internet Res. 2020 Dec 18;22(12):e19127. doi: 10.2196/19127 (PMC7775817; doi:10.2196/19127)
Supplement: Multimedia Appendix 3 [file jmir_v22i12e19127_app3.doc]

**Appendix 2 Data extraction form**

| Concept | Definition |
| --- | --- |
| Author | Study first author |
| Year | The year the study was published |
| Country | The country where the study was published |
| Publication type | The medium in which the study was published (conference abstract, journal article, dissertation) |
| Chatbot aim | What the study aimed to accomplish (education, therapy, diagnosing, counseling, ...) |
| Targeted Disorder | Disorder targeted by the chatbot |
| Chatbot name | Name of the chatbot |
| Platform | In the platform on which the chatbot is designed to operate (web-based, stand-alone software) |
| Dialogue language | The language by which the chatbot communicates with the user |
| Input modality | How the user interacts with the chatbot: text (via keyboards and mouse) voice (via microphones), non-verbal language (facial expression and body language via camera or Kinect). |
| Output modality | How the chatbot interacts with the user: text (via text on the screen), voice (via speakers), non-verbal language (facial expression and body language via embodiment). |
| Text understanding technology | Technology used to process and understand user input: Pattern Matching, Machine Learning, NLP, Web Service, Fixed Input, Rule Based, Hybrid |
| Text generation technology | Technology used to generate output for the user: fixed or machine generated output |
| Dataset used | The type of database that is stored and used by the chatbot: Medical knowledge database, User Information database, Conversation scripts, Not mentioned |
| Linking text generation and text understanding modules | How the text generation and text understanding modules are connected: Pattern matching Algorithms, Rule based methods, Machine learning, not mentioned, web based |
